# Equity as a Guiding Principle for the Public Health Data System

**DOI:** 10.1089/big.2022.0204

**Published:** 2022-09-06

**Authors:** Anita Chandra, Laurie T. Martin, Joie D. Acosta, Christopher Nelson, Douglas Yeung, Nabeel Qureshi, Tara Blagg

**Affiliations:** ^1^ RAND Corporation, Arlington, Virginia, USA.; ^2^ RAND Corporation, Santa Monica, California, USA.; ^3^ Pardee RAND Graduate School, Santa Monica, California, USA.

**Keywords:** equity, public health, data

## Abstract

The growing centering of equity in health has elevated a conversation about how those interests should translate within the systems and sectors that influence health. In particular, the public health data system has been relatively limited in capturing the drivers and consequences of health inequity as well as the varying dimensions of equity. This article examines what it means to use equity as a guiding principle throughout the components and functions of a modern public health data system. As with other articles in this supplement, this article builds from a literature review, environmental scan, and deliberations from the National Commission to Transform Public Health Data Systems to summarize current gaps to integrate equity throughout the system. It outlines opportunities for the technology and data science sectors specifically to engage given the access that these sectors have to information that would illuminate and frame the nuances and impacts of health inequity.

## Introduction

With the amplification that COVID-19 provided in illuminating health inequities, the issue of equity has come to the fore in public health discussions, including in analyses of how to transform the public health system.^1^ One of the critical challenges has been the ability of data within the public health system to not only highlight inequities but also help in the anticipation and ability to address drivers of those inequities. Given the primacy of health equity and the devastating consequences of inequity, it is argued that equity and equity orientation should not be a feature but must be the primary purpose of the modern public health data system going forward.

Health equity means that everyone has a fair and just opportunity to be as healthy as possible.^2^ This requires removing obstacles to health such as poverty, discrimination, and their consequences, including powerlessness and lack of access to good jobs with fair pay, quality education and housing, safe environments, and health care.^3^ However, it is unclear how equity should center in the modern public health data system, what it means to have an equity orientation, and why starting with equity offers a new roadmap for configuring a public health data system that is distinct from what we currently have. Further, with all the information we could have about individuals and communities and their health and well-being given advancements in data content and science, it begs questions about how this data modernization can embrace equity principles from the start.

In this article, we briefly set the stage for the equity orientation of the modern public health data system, the philosophical and pragmatic underpinnings of holding equity centeredness, and what this means for measurement, data collection, data analysis, and end use. We describe what has been missing from the equity ambitions of the current data system and what it will take to develop a more equity-centered data system. The equity challenge can be viewed as an opportunity for leaders in data science and technology, who hold critical information about health inequities and drivers of inequities. This could be brought to bear for the move toward an equity-centered, modern public health data system.

## Methods

In 2020, the Robert Wood Johnson Foundation formed the National Commission to Transform Public Health Data Systems to review significant challenges to the current public health data system, and “provide recommendations to policymakers, health care organizations and institutions, service providers, and philanthropy” on the potential solutions to overcome these challenges.^4^

In support of this effort, RAND conducted a supporting analysis that included an environmental scan to identify key issues, points of consideration, tradeoffs and tensions, and current activities related to public health data, data systems, and data modernization efforts. This effort included a targeted scan of published research papers and reports, reviews of websites and working documents describing coordinated activities (e.g., data interoperability), and recent initiatives. Additional searches included the use of “big data” in public health, data privacy, and ethics of public health data collection. Although the team primarily focused on public health data, it also identified seminal articles and reports from other sectors or disciplines whose findings could apply to public health data systems.

RAND simultaneously conducted semi-structured interviews with 112 experts and thought leaders on the main topics before the Commission. Individuals represented diverse sectors, including public health and health care, technology and data science, research and policy, journalism, and law. The interviews also included experts in data, data use, equity, community engagement, and research translation who work *outside* the traditional health sector. The project was reviewed and approved by the RAND Human Subjects Protection Committee.

In this article, we highlight relevant findings from this supporting analysis and then implications for the data science and technology sectors, with a consideration of recommendations that emerged from the final Commission report.

## Findings

When applying an *equity lens*^*†*^ to the purpose of public health data and the public health data system, significant questions emerged from both literature and stakeholder inputs regarding whether equity is honestly reflected in the public health data system's organizing mission. For instance, the goal of Healthy People 2030 is to improve the health and well-being of people in the United States, with underlying objectives pointing to the need to reduce disparities and achieve health equity as part of this goal.

These kinds of objectives are not uncommon in public health, and their importance has certainly been underscored by recent discussions elevating health inequities as untenable, particularly considering COVID-19.^5–7^ Although the Healthy People objectives and others like them are laudable, there is a challenge related to equity. Is health equity truly a throughline of a national scorecard like this, such that equity is understood as both process and outcome? Are the components of equity, such as procedural and distributive equity, assessed and to what extent are drivers of inequity, such as history, cumulative stresses, and structures, monitored as part of the public health data system?

In short, insight from stakeholders and the current landscape revealed that the modern public health data system must be able to capture all forms of equity, and yet currently falls short.^2^

There are several types of equity, but rarely have these aspects of equity been explicitly acknowledged in the choice of public health data and the design of the public health data system.^2^

Equity orientation in the public health data system must outline how the parameters of equity are set (e.g., who is included in decision making, who are the targets of the effort in terms of *for whom* to improve equity); why equity is the focus (vs. a “do no harm” model, for instance, that does not consider differential population needs and histories); who counts as the subject of equity and how are generational and historical considerations taken into account; and then given that, what is the content of the equity (i.e., what counts as a matter of equity). This includes^8^:

- Procedural equity: the perceived fairness of processes and procedures to make decisions;
- Distributive equity: how social welfare and need is balanced; and
- Contextual equity: how pre-existing social conditions influence equity.

We talk about each type of equity and the current gaps in the following sections. We then turn to the implications for those in the data science and technology sector.

### A lack of procedural equity has impeded trust between the public and public health leaders

Procedural equity addresses how the concept of fairness is included in approaches and policies, once the equity parameters are set (i.e., for whom are we improving equity).^8^ People must believe in the justness of the system, with attention to trust, ethics, voice, and participation. The issue of trust in public health is well documented as influencing everything from adherence to medication regimes and behavioral change interventions to the uptake of health risk communications.^9^ We know that transparency and engagement of the public in decision making can be helpful antidotes to trust concerns, particularly in the times of uncertainty that a pandemic or other emergent threat presents.^10^

This type of procedural equity steps beyond trust to include components such as representation, as well as voice and participation in decision making. In the context of public health data, these components are meaningful, yet not systematically pursued.^2^ Representation and inclusion have become even more resonant during COVID-19, stemming from a lack of granularity in key demographic categories, such as race/ethnicity or disability status, hampering an ability to respond effectively to the needs of certain subgroups.

The issue of voice and participation in decision making is a persistent problem in public health, and the public health data system is no exception. By voice,^11^ we reference the inclusion of the perspectives, ideas, and lived experiences of those impacted by public health decisions. By participation,^12^ we reference the active role of those impacted by public health decisions in the actual process of arraying decision options and providing meaningful input on those decisions.

There is growing evidence about the benefits of having people involved in health decision making because the health field confronts difficult tradeoffs of resources and values, and values are often driven by ethics and social and cultural experiences.^12^ And yet despite this recognition, full public participation in public health decision making is not common. This problem has been particularly acute among those groups that have been historically marginalized and disproportionately affected by health issues and negative health exposures.^13^

If there were full voice and participation in decision making in the modern public health data system, this would include meaningful inputs on measures selection, how data are collected, how data are disaggregated, how data are represented, and how information and insight are drawn from the data.^14^ Ideally, with more attention to procedural equity, data or digital colonialism, a concern that the private sector is harvesting data without reciprocal public benefit, could be less of an issue.^15^

### Without distributive equity represented in the public health data system, it is difficult for the data system to fully support its sense-making *(or the cognitive processes by which people make meaning from data and experiences)* and decision-making functions

The concept of distributive equity focuses on allocation and resource management decisions, with attention to the balance of costs, risks, and benefits.^8^ Usually, distributive equity considers how decisions are made and benefits are distributed based on the dimensions of need and social benefit.

It is unclear how much the concepts of distributive equity are embedded in the design of the current public health data system, but as the modern public health data system grapples with how to center equity and how to support forward-leaning public health action, it is useful to examine which data are used and how data are arrayed to inform allocation decisions. To date, many of the public health data dashboards organize information into some combination of dimensions of clinical outcomes, the social and economic environment, and risk behaviors.

But most datasets and data platforms do not organize data in ways that align measures and indicators in a distributive equity framework.^2^ For instance, this can mean organizing data to cluster indicators about the proximal drivers (e.g., insurance status, quality of health services, prenatal care, discrimination) of a particular health outcome (e.g., maternal mortality) with the health outcome so that decision makers are considering the investments needed in those drivers together to influence an outcome, and what is then possible given resource constraints.^16–19^ A transformed public health data system could offer information and, ultimately, insights, which help with tradeoffs for realizing concepts, such as targeted universalism and efforts to address historical inequities.

### Contextual equity regarding pre-existing social conditions and its influence on equity is a significant gap

Contextual equity is the backdrop of both procedural and distributive equity, because this form of equity accounts for the political, economic, social, and intergenerational factors in which populations engage with society, its systems, and its benefits. This includes contextual variables such as access (e.g., access to capital) and power (e.g., in this context, the ability to gain and maintain access to resources).^8^

Despite its importance, in the context of the public health data system, contextual equity has tended to receive less attention both in the content and type of data, which data are tracked, and how those data are translated into public health action. It is rare that variables such as the accumulation of risk exposures, the legacy of injustice, and systemic barriers are factored into how measures of health are calculated or how data are used to justify or explain certain types of public health action.

Without intentional pursuit of information about pre-existing inequities, it is difficult to take typical public health data and use it in a way that can lead to equitable action, particularly over time and across generations. As such, transforming public health data must include consideration of how the translation of data into information and insight has a historical explanation included in the interpretation.^2^

## Implications

During the stakeholder analysis, review of existing data systems and dashboards, and Commission deliberations, it not only became clear that the current public health data system falls short in representing the comprehensive dimensions of equity, but also that many sectors must be part of realizing the equity-centered expectations of the next-generation public health data system.^2,4^ In short, this cannot be solved through a governmental public health lens alone.

Capturing the complexities of health inequity—whether the intersectionalities of health impacts by overlapping population characteristics or the ability to contextualize why those health inequities have emerged—will require new forms of data and new ways to merge data, visualize those data, and tell stories from the data to drive data sense-making and decision making. Specifically, technology companies, with access to information about community context and historical patterns, and data scientists, with analytic tools to connect seemingly disparate, cross-sector drivers of health inequity, are needed to advance procedural, distributive, and contextual equity.

There are a few areas that are ripe for stronger data science and tech sector engagement. For instance, one of the Commission's recommendations was squarely focused on equity by asking technology companies to support public health data system transformation in under-resourced areas of the country with the largest health inequities, by either direct financial support or indirect collaborations.^4^ Further, the Commission argued that public health innovation could be fostered by promoting methods^4^; some of those methods are key to understanding equity.

We highlight two major implications for this sector here.

### Data science and technology companies can help to augment transparency and power in data access, use, and decision making

Equity requires participation and justice, and there have been challenges to those themes when considering topics such as “big data” and algorithmic bias. As noted in the role of procedural equity, the issue of representation in health decision making must be a key part of a modern, equity-oriented public health data system, but there remain concerns about the extent of participation, accountability, and power^19^ (or the authority to shape expectations, decisions, and outcomes in public health).

The current public health data system is not usually characterized by transparency, support of data access and use that facilitates sustained civic engagement, and deep consideration of the voices of historically marginalized and chronically underrepresented populations.^2^ This focus on equity in data use and decision making is even more critical in the context of the greater volume, velocity, and variety of data (characteristics of “big data”), the role of technology in data generation and manipulation (e.g., artificial intelligence, machine learning), and increasing challenges to civic engagement and democracy. Kalluri wrote, “Don't ask if AI is good or fair, ask it if it shifts power.”^20^

This sentiment targeting AI was focused on ensuring that the tool does not exacerbate inequities through problems such as algorithmic bias, but this theme can also be widened and attributed to all the components of a modern public health data system. In discussions with stakeholders about the future of public health data, there was an overriding consensus that the power in the public health data system was not oriented properly.^2^ Further, data were not “righting” social inequities but rather potentially worsening those inequities by aligning data in ways that do not support equity-promoting policy solutions.

In the context of new forms of data or “big data,” these issues of participation and representation are even more resonant.^21^ Key ethical issues meriting more comprehensive engagement include fair distribution of benefits and burdens, control and sharing of data, and accountability. For instance, people participation is needed to determine whether the benefits of these “big data” accrue to corporations while the burden is borne by individual citizens. How private companies hold information as business assets versus supporting community benefit is a key consideration. Further, how data are used for good, whose good, and then how data can be used for harm also is a question of accountability to whom and by whom.^22^

### Data science and technology companies can expand their partnership with public health agencies and other public health leaders in informing the public health and equity narrative

In addition to this issue of what is collected and analyzed and how those most impacted are included in data sense-making, there is the topic of data story. Communicating the intricacies of equity and its role in health outcomes is not an easy task. The ability to explain the impacts of systemic and structural racism requires analytic storytelling, and other tools that quite simply are not often in the resources or realm of governmental public health. Given this, there are two clear roles for the data science and technology sector going forward.

One is to provide supporting platforms and bridges to other public health actors in communities to aid in explaining the historical links of racism and other inequities to health outcomes today, in ways that ensure the public can both understand the information and apply it to redress distributive equity issues. The illumination of disparities only (i.e., just describing gaps between groups) has left public health falling short, but information that explains the drivers of health inequity can aid local response by tying those inequities to policies and other paths for intervention.

The second area for the data science and tech sector is the sustained engagement and training of the public health workforce in how to center equity in the design of data platforms, such that these stories of historical and current inequity can be explained. This means revisiting how data dashboards are organized to allow for contextual equity considerations, and how those in data science and technology learn to communicate those findings in ways that can lead to social change and structural action.

## Conclusion

Although the task of public health data transformation is daunting and requires a level of cross-sector collaboration often not observed, the opportunities for the data science and tech sector to engage in positive ways to address health equity are clear. Moving ahead, more operational discussions about equity-centered data and representation will require new terms of collaboration and data governance that elevates public and private sector cooperation to a new level of engagement.

## Notes

^†^
An **equity lens** is defined as any approach for analyzing the equity impact of actions on people or places, with attention to those historically marginalized, as well as the process of addressing any structural or systemic barriers to the opportunity to be healthy (*adapted from multiple sources*).
